# Human T-cell leukemia virus type-I Tax induces the expression of CD83 on T cells

**DOI:** 10.1186/s12977-015-0185-1

**Published:** 2015-07-01

**Authors:** Yuetsu Tanaka, Mariko Mizuguchi, Yoshiaki Takahashi, Hideki Fujii, Reiko Tanaka, Takuya Fukushima, Takeaki Tomoyose, Aftab A Ansari, Masataka Nakamura

**Affiliations:** Department of Immunology, Graduate School of Medicine, University of the Ryukyus, 207 Uehara, Nishihara-cho, Okinawa, 903-0215 Japan; Human Gene Sciences Center, Tokyo Medical and Dental University, 1-5-45 Yushima, Bunkyo-ku, Tokyo, Japan; Laboratory of Hematoimmunology, School of Health Sciences, Faculty of Medicine, University of the Ryukyus, Okinawa, Japan; Division of Endocrinology, Diabetes and Metabolism, Haematology, Rheumatology (Second Department of Internal Medicine), Graduate School of Medicine, University of the Ryukyus, Okinawa, Japan; Department of Pathology, Emory University School of Medicine, Atlanta, GA USA

**Keywords:** CD83, HTLV, Tax, ATL, PGE2

## Abstract

**Background:**

CD83, a cell surface glycoprotein that is stably expressed on mature dendritic cells, can be transiently induced on other hematopoietic cell lineages upon cell activation. In contrast to the membrane form of CD83, soluble CD83 appears to be immunosuppressive. In an analysis of the phenotype of leukemic CD4^+^ T cells from patients with adult T-cell leukemia (ATL), we found that a number of primary CD4^+^ T cells became positive for cell surface CD83 after short-term culture, and that most of these CD83^+^ CD4^+^ T cells were positive for human T-cell leukemia virus type-I (HTLV-I) Tax (Tax1). We hypothesized that Tax1 is involved in the induction of CD83.

**Result:**

We found that CD83 was expressed selectively on Tax1-expressing human CD4^+^ T cells in short-term cultured peripheral blood mononuclear cells (PBMCs) isolated from HTLV-I^+^ donors, including ATL patients and HTLV-I carriers. HTLV-I-infected T cell lines expressing Tax1 also expressed cell surface CD83 and released soluble CD83. CD83 can be expressed in the JPX-9 cell line by cadmium-mediated Tax1 induction and in Jurkat cells or PBMCs by Tax1 introduction via infection with a recombinant adenovirus carrying the Tax1 gene. The CD83 promoter was activated by Tax1 in an NF-κB-dependent manner. Based on a previous report showing soluble CD83-mediated prostaglandin E2 (PGE2) production from human monocytes in vitro, we tested if PGE2 affected HTLV-I propagation, and found that PGE2 strongly stimulated expression of Tax1 and viral structural molecules.

**Conclusions:**

Our results suggest that HTLV-I induces CD83 expression on T cells via Tax1 -mediated NF-κB activation, which may promote HTLV-I infection in vivo.

**Electronic supplementary material:**

The online version of this article (doi:10.1186/s12977-015-0185-1) contains supplementary material, which is available to authorized users.

## Background

CD83 is a 40–50-kDa cell surface glycoprotein that is a member of the sialic-acid-binding immunoglobulin-like lectin family, and is a marker of mature dendritic cells (DCs) in humans and mice [1]. In contrast to mature DCs, which stably express high levels of CD83 [2], previous studies suggest that CD83 is transiently expressed on other hematopoietic cell lineages and tissues, including activated T cells and B cells [1, 3–5], macrophages [2], neutrophils [6], and NK cells [7] in vitro, and hematopoietic tissues [8] and skin Langerhans cells [1] in vivo.

Two isoforms of CD83, a membrane-bound form (mCD83) and a soluble form (sCD83) have been identified; sCD83 is likely a result of shedding the mCD83 isoform [9]. mCD83 critically functions not only in the development of CD4^+^ T cells in the thymus [8], but also in T cell activation [10] and the longevity of B and CD4^+^ T cells [4]. In contrast, sCD83 functions as a suppressor of T cell activation [11]. A recent study has shown that sCD83 suppresses the differentiation of DCs from monocytes [12]. Elevated levels of plasma sCD83 have been demonstrated in patients with hematological malignancies [13]. Kreiser et al. showed that the in vitro culture of mCD83-expressing fresh regulatory T cells in mice leads to the release of sCD83 [14]. Although the molecular mechanisms by which sCD83 mediates T cell suppression are not fully understood, human sCD83 may mediate its inhibitory effect on T cell responses via prostaglandin E2 (PGE2) produced by monocytes [15].

Human T-cell leukemia virus type-I (HTLV-I) is a human retrovirus that is etiologically associated with adult T-cell leukemia (ATL) [16] and HTLV-I-associated myelopathy/tropical spastic paraparesis (HAM/TSP) [17, 18]. The majority of HTLV-I carriers remain asymptomatic throughout their lives, and approximately 5% of HTLV-I-infected individuals develop either ATL or HAM/TSP after prolonged latency periods [19]. HTLV-I encodes Tax (Tax1), which activates viral transcription and promotes mechanisms that are critical for cell growth and division, leading to viral replication [20]. The effects of Tax1 on cells include potent NF-κB activation, cell cycle perturbation, and cell transformation [21]. In addition to Tax1, the basic leucine zipper of HTLV-I (HBZ) has been proposed to play an important role in the oncogenesis of ATL by HTLV-I [22]. However, the precise mechanisms by which HTLV-I infection leads to disease development remain poorly understood.

HTLV-I is transmitted through contact with body fluids containing infected cells; common examples include mother-to-child transmission [19]. It is difficult to detect HTLV-I-antigen-expressing cells in fresh PBMCs from HTLV-I infected individuals. When PBMCs are cultured for a short period of time, some T cells begin to produce HTLV-I antigens [23] followed by spontaneous immortalization of cells in cultures containing interleukin-2 (IL-2) [24]. CD8^+^ cytotoxic T lymphocyte (CTL) [25] and neutralizing antibody [26] responses specific to HTLV-I play important roles in controlling HTLV-I propagation in HTLV-I carriers. It is noteworthy that not only ATL patients, but also healthy HTLV-I carriers are prone to immunodeficiency [27–31]. Curiously, monocytes from HTLV-I^+^ donors exhibit a deficiency in the ability to differentiate into mature DCs in vitro, and cultured immature DCs show low levels of CD83 expression in association with poor T cell stimulation [32].

In an analysis of phenotypes of PBMCs from ATL patients and HTLV-I carriers living in Okinawa prefecture, Japan, where ATL is prevalent, we detected high levels of CD83 expression in primary CD4^+^ T cells from PBMCs of HTLV-I^+^ donors after short-term in vitro culture. Surprisingly, most of these CD83^+^ cells from HTLV-I^+^ donors were positive for Tax1. Herein, we show that Tax1 is responsible for the induction of CD83 on T cells, and discuss its possible role(s) in HTLV-I infection.

## Results

### CD83 expression on Tax1^+^ cells

**Figure 1:**
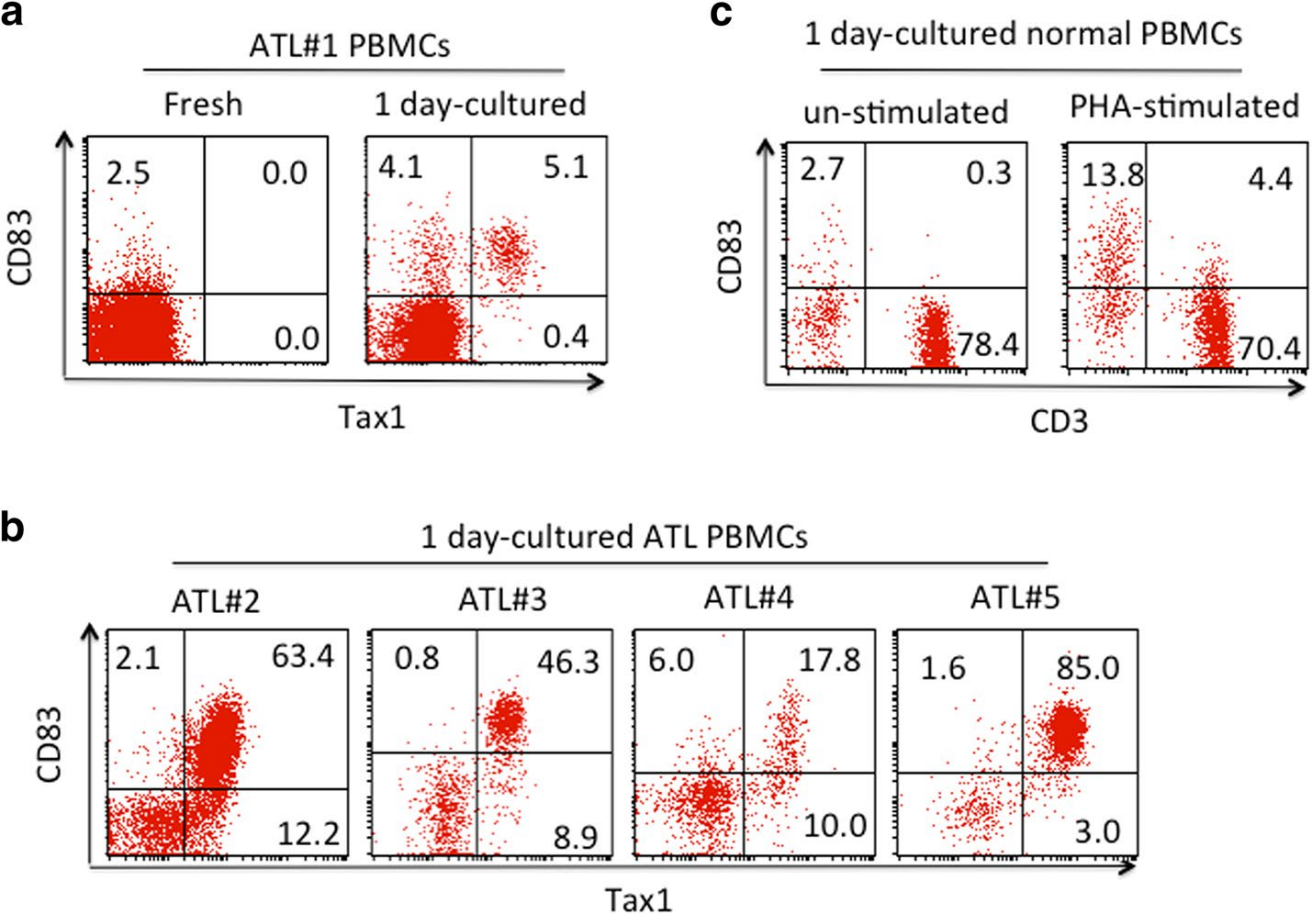
Flow cytometric analysis (FCM) of CD83 expression on primary CD4^+^ T cells expressing Tax1. **a**, **b** Lymphocytes were gated based on FSC and SSC in peripheral blood mononuclear cells (PBMCs) derived from adult T cell leukemia (ATL) patients (ATL# 1 to #5) and examined for expression of CD83 and Tax1 before or after a 1-day culture. Most ATL patient-derived PBMCs consisted of leukemic CD4^+^ T cells (data not shown). **c** Lymphocytes in PBMCs from normal donors were examined for CD83 expression after a 1-day culture in the presence or absence of phytohemagglutinin (PHA). Representative profiles are displayed.

In a phenotypic analysis of fresh and in vitro cultured PBMCs from ATL patients in Okinawa, we noticed that short-term cultivation induced CD83 expression in a subpopulation of CD4^+^ T cells in PBMCs (Figure 1a, b). Interestingly, a majority of the CD83-positive CD4^+^ T cells exhibited detectable levels of intracellular Tax1 expression. The Tax1 expression in cultured PBMCs from ATL patients was confirmed by a western blot analysis with our anti-Tax1 monoclonal antibody (mAb) (Lt-4) (Additional file 1: Figure S1). Similar acquisition of CD83 on Tax1^+^ cells was observed in cultured PBMCs from HTLV-I carriers and HAM/TSP patients (data not shown). In contrast, only low levels of CD83 expression were observed in normal PBMCs cultured in vitro without mitogen. Cultures with mitogen exhibited increased CD83 expression in a small population of CD3^-^ PBMCs (Figure 1c), which were predominantly CD19^+^ (data not shown), indicating that those CD83^+^ cells were B lymphocytes.

**Figure 2:**
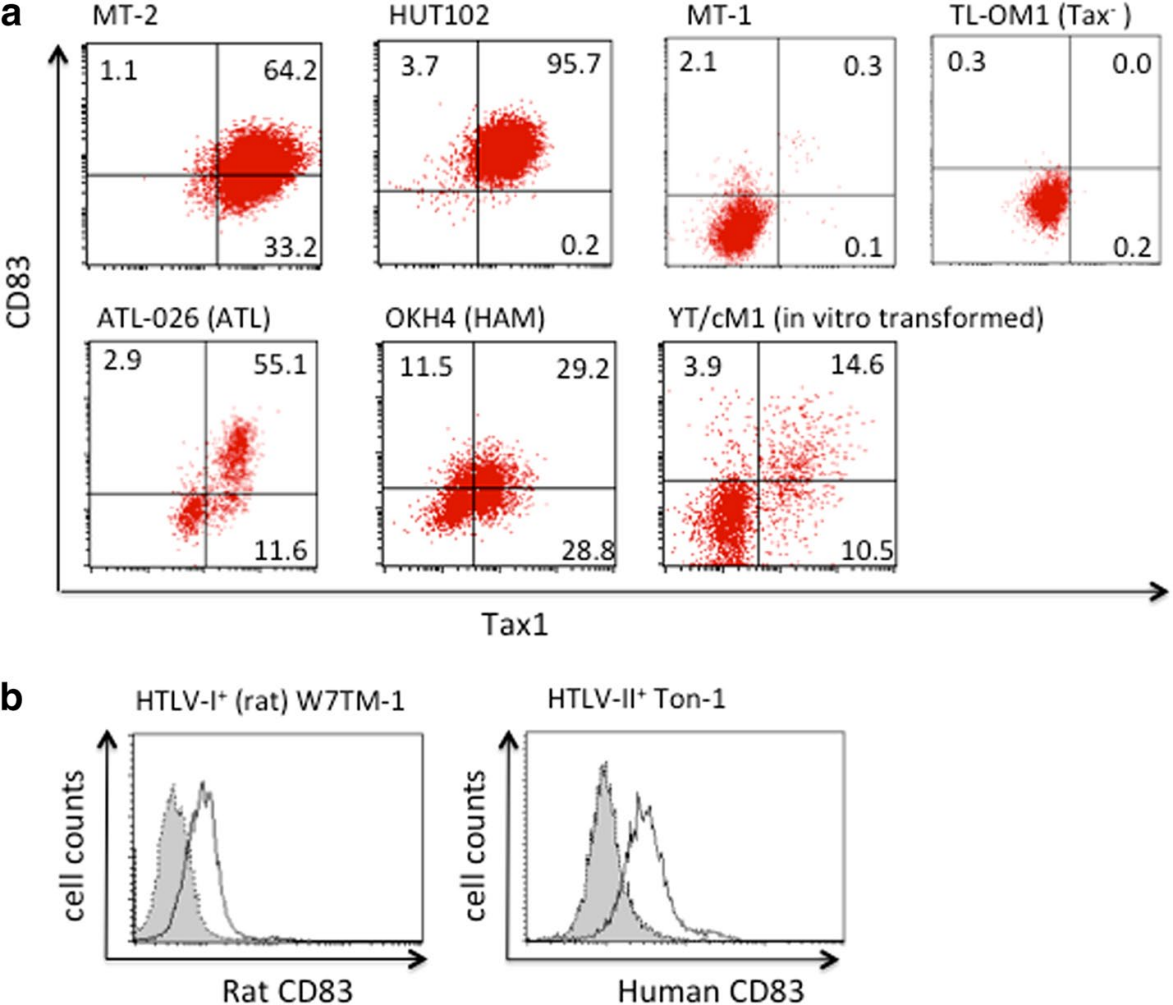
CD83 expression on HTLV-I^+^ and HTLV-II^+^ T-cell lines. **a** HTLV-I^+^ T cell lines (MT-2, HUT102, MT-1, and TL-Om1), and IL-2-dependent T cell lines generated from an ATL patient (ATL-026, CD4^+^), a HAM/TSP patient (OKH4, CD8^+^), and a normal donor (in vitro-transformed YT/cM1, CD4^+^) were examined for CD83 and Tax1 expression by FCM. Note that the TL-Om1 cell line is negative for Tax1. **b** Rat and human CD83 expression levels on the rat CD4^+^ Tax1^+^ T cell line (W7TM-1) and the human HTLV-II^+^ T cell line (Ton1), respectively, were analyzed by FCM.

We next examined CD83 expression with a panel of HTLV-I^+^ T cell lines. All of the Tax1^+^ cell lines tested, including the IL-2-independent T cell lines (MT-2, HUT102, and MT-1) and IL-2-dependent T cell lines derived from an ATL patient (ATL-026), a HAM/TSP patient (ILT-M1), or a healthy donor whose T cells were transformed in vitro by HTLV-I (YT/cM1), were positive for CD83 (Figure 2a). It is noteworthy that an ATL patient-derived HTLV-I-infected T cell line that lacks Tax1 expression (TL-Om1) was negative for CD83 expression. In addition, the HTLV-I^+^ rat T cell line W7TM-1, and the human HTLV-II^+^ T cell line Ton1 were also positive for rat and human CD83, respectively (Figure 2b). The HTLV-I-negative human T cell lines CEM, Molt-4, and Jurkat and the HIV-1-producing Molt4/IIIB were negative for CD83 (data not shown). CD83 expression thus appeared to parallel the expression of Tax1. These results suggest a relationship between CD83 and Tax1 expression.

**Figure 3:**
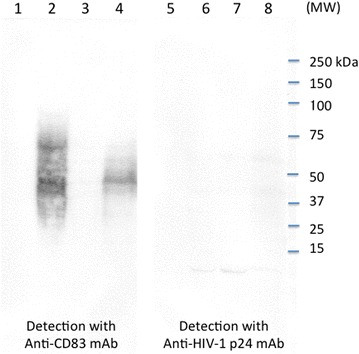
Western blot analysis of CD83 expressed in the HTLV-I^+^ T cell line HUT102. Cell lysates from control 293T/CT (*lanes 1* and *5*), CD83-transfected 293/TCD83 (*lanes 2* and *6*), CEM (*lanes 3* and *7*), and HUT102 (*lanes 4* and *8*) were subjected to sodium dodecyl sulfate polyacrylamide gel electrophoresis (SDS-PAGE) on a 5–20% gel, and blotted onto PVDF membranes. The membranes were incubated with either rat anti-human CD83 monoclonal antibody (mAb) (clone W#83-8, *lanes 1–4*) or isotype control rat IgG (clone WAP-24,* lanes 5–8*) antibody, followed by treatment with HRP-labeled goat anti-rat IgG antibody.

The expression of CD83 in HTLV-I^+^ cells was confirmed by western blot analyses using our laboratory-derived mAb (rat IgG, clone W83#8). As shown in Figure 3, the apparent molecular weight (MW) of the major CD83 molecule in HTLV-I^+^ HUT102 cells was 48 kDa, which was comparable to that of ectopically expressed CD83 on 293T cells (293T/CD83). Since the protein core of CD83 has an estimated MW of 21 kDa and is heavily glycosylated [9], we inferred that the other CD83-specific bands ranging between 40 and 70 kDa may be CD83 isoforms with different levels of glycosylation.

**Figure 4:**
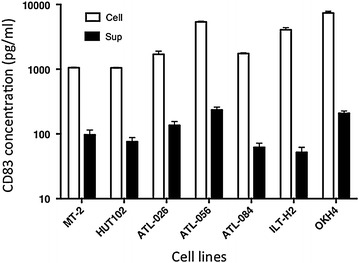
Quantitation of cell-associated CD83 and sCD83. The HTLV-I^+^ T cell lines (MT-2 and HUT-102) and T cell lines from either ATL patients (ATL-026, ATL-056, ATL-084, ILT-H2) or a HAM/TSP patient (OKH4) were cultured in a volume of 1 ml/well for 3 days in vitro. Cells and culture supernatants were separated by centrifugation, and the cells were lysed with 1 ml of a lysing buffer. The levels of CD83 in the culture supernatants or in the cell lysates were determined by ELISA. Results are shown as the mean ± SD of CD83 of triplicate cultures.

We next examined the production of sCD83 by HTLV-I^+^ T cells. Levels of CD83 in cell lysates and culture supernatants were quantitated using a commercial enzyme-linked immunosorbent assay (ELISA) kit. All Tax1^+^ T cell lines tested produced readily detectable levels of sCD83 in culture supernatants (Figure 4). The relative levels of sCD83 were estimated at approximately 10–20% of CD83 levels associated with cells. Since plasma sCD83 is elevated in some patients with chronic lymphocytic leukemia [33], we attempted to determine the levels of sCD83 in plasma samples obtained from acute ATL patients. Out of 11 ATL samples, only one showed a significantly higher sCD83 level (>1,000 pg/ml) than normal (<20 pg/ml) (data not shown).

### CD83 induction by Tax1

**Figure 5:**
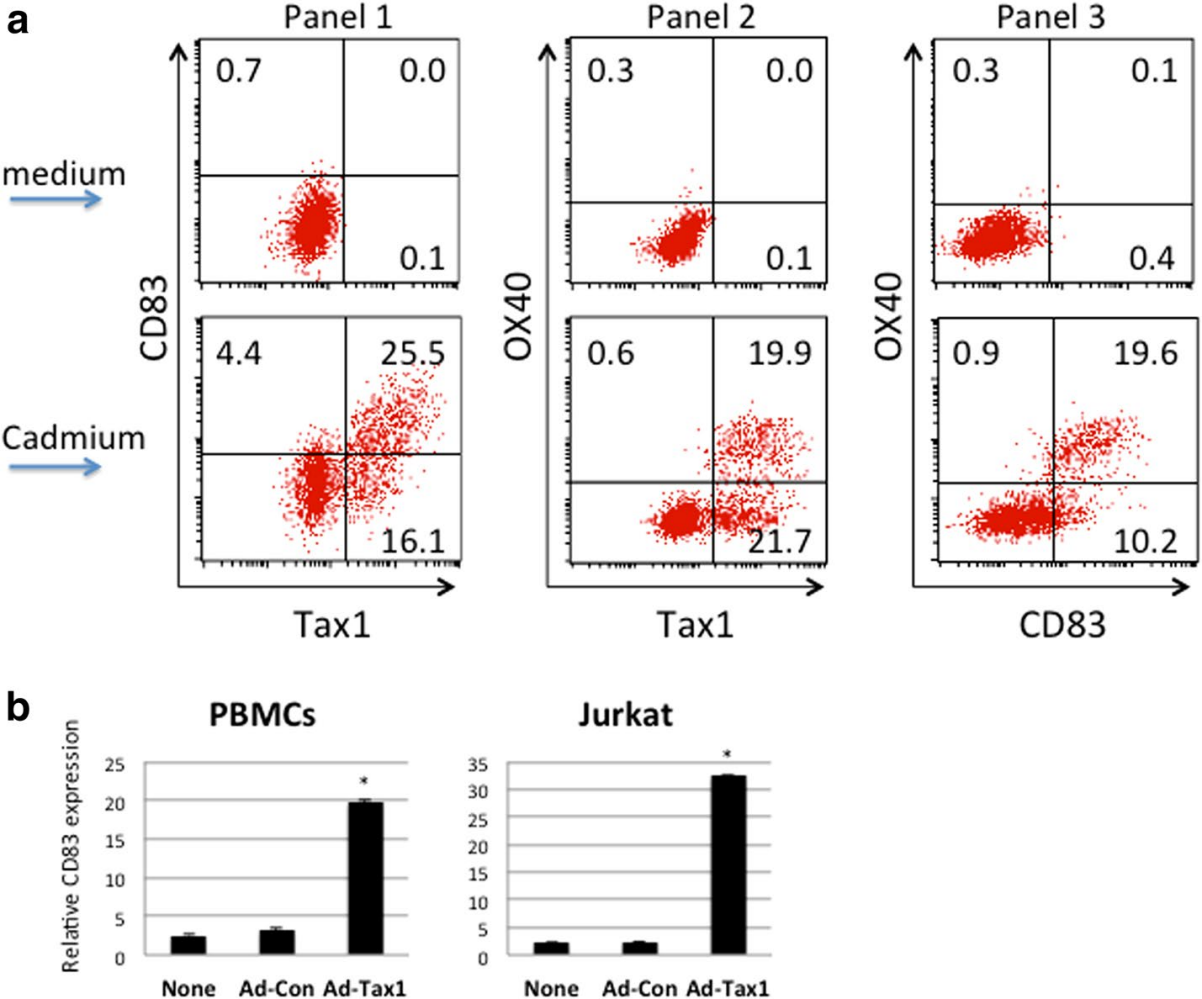
Induction of CD83 expression by Tax1. **a** CD83 and Tax1 co-expression (*Panel 1*), OX40 and Tax1 co-expression (*Panel 2*), and OX40 and CD83 co-expression (*Panel 3*) in JPX-9 cells were examined by FCM. Cells were cultured either in medium alone (*upper columns*) or in the presence of 10 μM CdCl_2_ (*lower columns*) for 3 days. **b** PBMCs and Jurkat cells were infected with recombinant Tax1 adenovirus (Ad-Tax1) or wild-type virus (Ad-Con), cultured for 48 h and harvested for mRNA isolation. The levels of CD83 mRNA were measured by quantitative PCR with specific primers indicated in the Methods section. The results are shown as the mean ± SE after normalization against 18S rRNA.

The potential ability of Tax1 to induce CD83 expression was examined using the Tax1-inducible human T cell line JPX-9. Incubation with CdCl_2_, which is required for Tax1 expression, induced apparent cell surface CD83 expression on JPX-9 cells (Figure 5a). Importantly, CD83 expression was predominantly restricted to Tax1^+^ cells. The majority of Tax1^+^ cells also expressed OX40, a representative protein that is also induced by Tax1 on T cells [34].

To confirm the Tax1-mediated CD83 expression, CD83 mRNA levels were quantitated by real-time PCR using peripheral blood mononuclear cells (PBMCs) and Jurkat cells following infection with recombinant adenovirus encoding Tax1 (Figure 5b). CD83 mRNA levels were significantly elevated in both PBMCs and Jurkat cells. These results are consistent with the concept that Tax1 induces cell surface expression of CD83, and suggest that the effect of Tax1 on CD83 expression occurs at the transcriptional level. Taken together, these data strongly imply that CD83 is a member of the HTLV-I Tax-inducible protein family.

### Enrichment of live Tax1^+^ and Tax1^−^ cells by cell sorting

**Figure 6:**
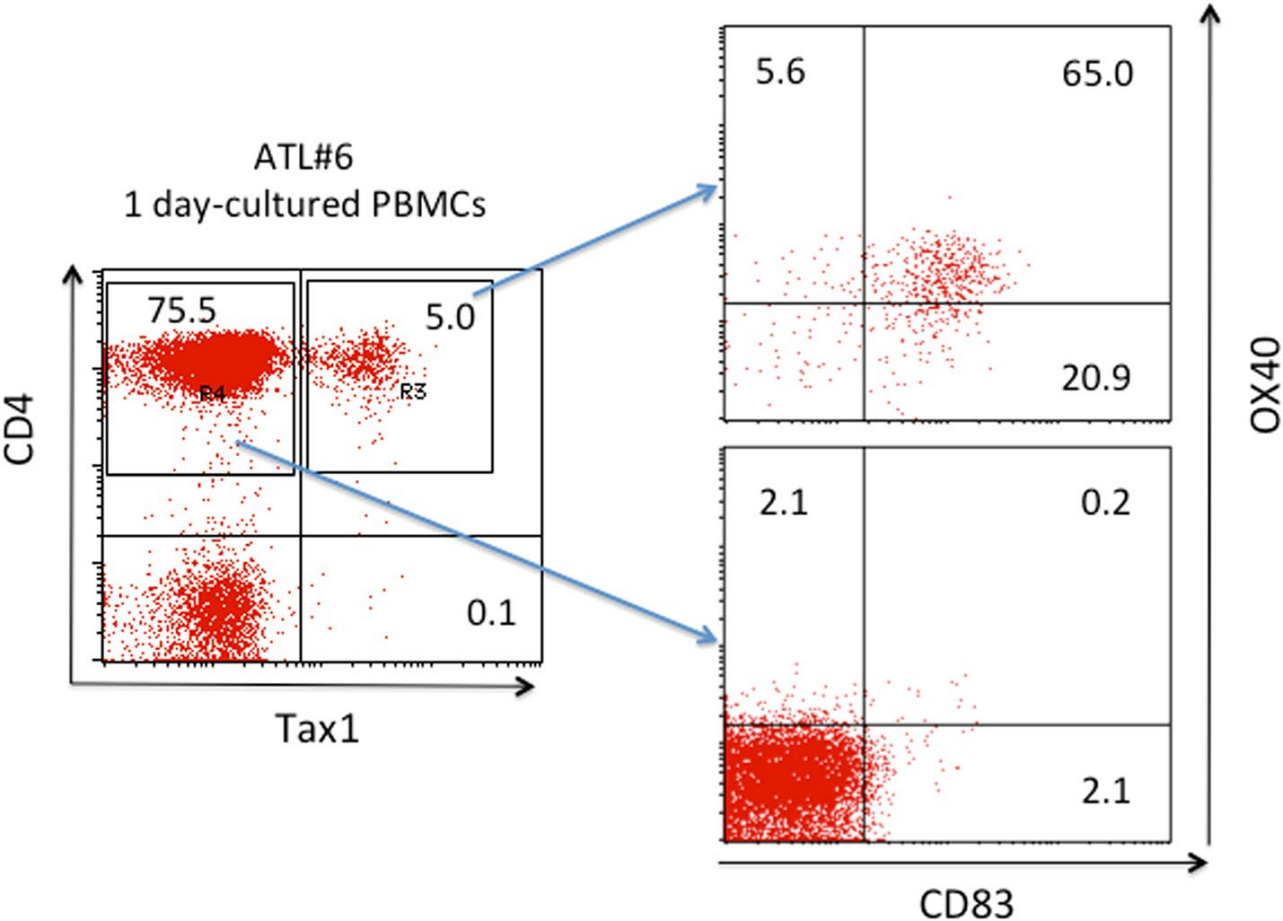
Co-expression of CD83 and OX40 on Tax1^+^ T cells. PBMCs of an ATL patient (#6) were cultured for 18 h and stained with antibodies against CD4, Tax1, CD83 and OX40. CD4^+^ Tax1^−^ cells and CD4^+^ Tax1^+^ T cells were gated and examined for their expression of CD83 and OX40 by FCM.

**Figure 7:**
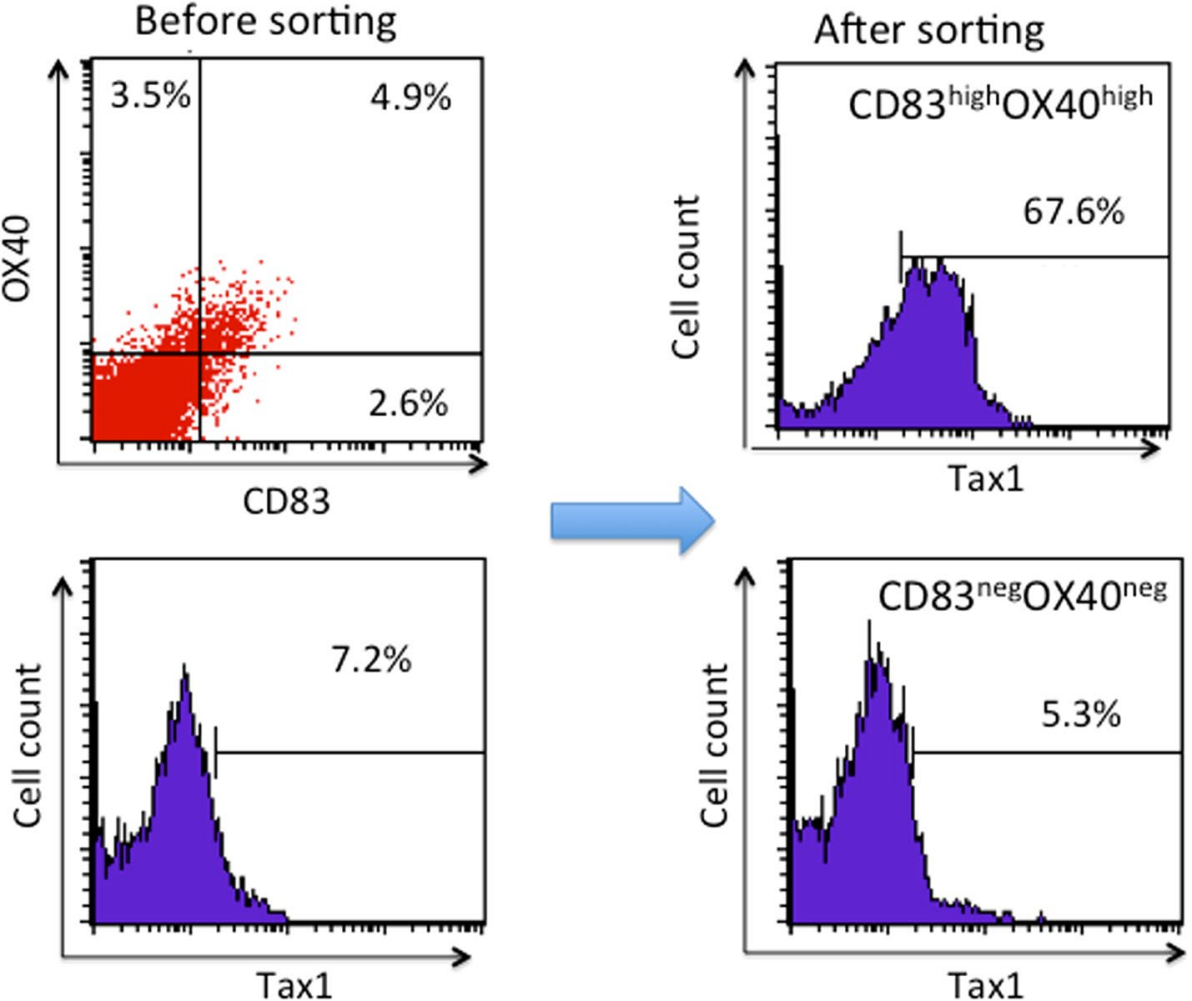
Flow cytometric analysis of Tax1 expression of cell sorter purified CD83^hi^/OX40^hi^ and CD83^negative^/OX40^negative^ populations. An IL-2 dependent HTLV-I^+^ T cell line generated from a HAM/TSP patient (OKH4) was stained with anti-OX40 and anti-CD83 mAbs. The CD83^+^OX40^+^ and CD83^−^OX40^−^ populations were sorted, and stained for Tax1. The Tax1 expression profiles prior to and post sorting are shown.

It may be noteworthy that the triple-positive (Tax1^+^ CD83^+^ OX40^+^) phenotype was found in primary CD4^+^ T cells from an ATL patient (Figure 6). Generally, IL-2-dependent HTLV-I-infected T cell lines derived from HTLV-I^+^ donors consist of HTLV-I antigen-positive and -negative cells, especially during the early culture stage with low passages. Flow cytometry-based cell sorting cannot separate live Tax1^+^ cells from live Tax1^−^ cells owing to intracellular localization of Tax1. Based on the present finding that most Tax1^+^ cells expressed both CD83 and OX40, we attempted to sort live Tax1^+^ and Tax1^−^ cells. An IL-2 dependent HTLV-I^+^ T cell line (OKH4) from a HAM/TSP patient was stained with anti-OX40 and anti-CD83 mAbs, and subjected to cell sorting. CD83^+^ OX40^+^ sorting efficiently enriched the Tax1^+^ cell population (Figure 7). Similar enrichment of Tax1^+^ cells was obtained with three IL-2-dependent HTLV-I^+^ T cell lines (data not shown). This strategy would be useful for further studies on Tax1 function in pHTLV-I-infected primary T cells.

### Tax1-responsive elements in the CD83 promoter

**Figure 8:**
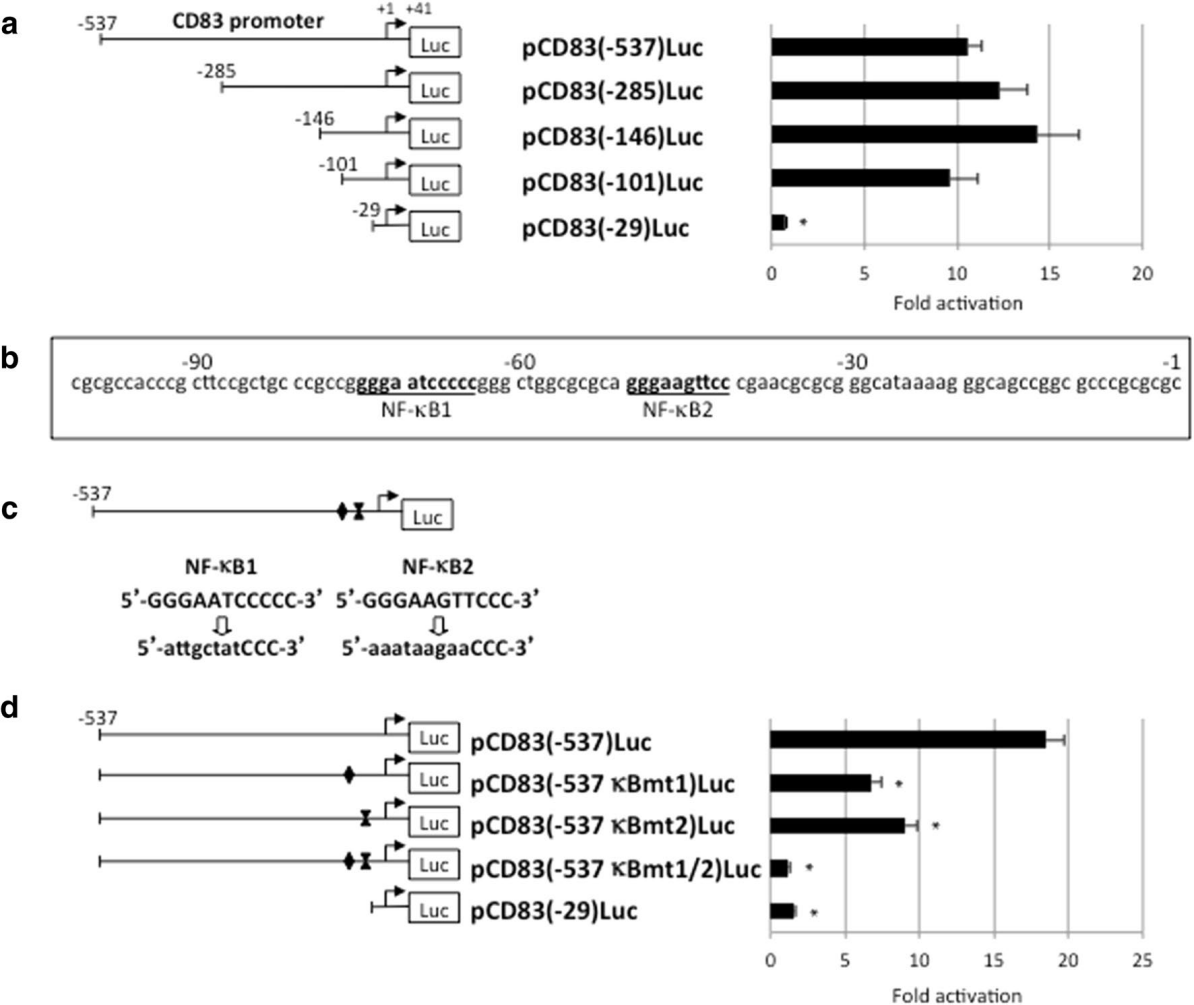
Tax1-responsive elements in the CD83 promoter. **a** Luciferase reporter plasmids carrying the wild-type (−537 to +41) and its 5′ deletion mutants (−285 to +41, −146 to +41, −101 to +41, and −29 to +41) of the CD83 promoter were transfected into Jurkat cells along with the Tax1 expression plasmid (pMT-2Tax). Cells were cultured for 48 h and analyzed for luciferase activity. The results were normalized based on protein content. **b** Nucleotide sequences of the CD83 promoter (−101 to −1 with respect to the transcription start site). Possible NF-κB binding sites (NF-κB1 and NF-κB2) are underlined. **c** Schematic representation of the possible NF-κB binding sites in the CD83 promoter is shown with lowercase letters indicating mutated sequences. **d** Reporter plasmids carrying the wild-type CD83 promoter or mutant promoter were transfected into Jurkat cells along with the Tax1 expression plasmid (pMT-2Tax). Cells were cultured for 48 h and analyzed for luciferase assay. The results shown are as the mean ± SE after normalization based on protein content.

To gain insight into the molecular mechanism of Tax1-dependent induction of CD83, the CD83 promoter was examined by reporter assays. Jurkat cells were transfected with a luciferase reporter plasmid, pCD83(-537)Luc, carrying the isolated wild-type CD83 promoter along with the Tax1 expression plasmid. The CD83 promoter was profoundly activated by Tax1 (Figure 8a). In efforts to localize the CD83 promoter sequences with biological activity, reporter assays were performed using a series of 5′ deletion constructs of the CD83 promoter. These studies showed that pCD83(-101)Luc retained activity to promote transcription in response to Tax1, similar to pCD83(-537)Luc, while pCD83(-29)Luc did not respond to Tax1 (Figure 8a). The observations indicate that a Tax1-responsive element(s) in the CD83 promoter is located in the region between −101 and −30. A computer search identified two possible NF-κB binding sites between the −101 to −30 region of the CD83 promoter (Figure 8b). We next investigated the potential role of those NF-κB binding sites in Tax1-mediated activation of CD83 gene transcription. This objective was achieved using substitution mutations that were introduced to either each of the possible NF-κB binding sites or to both sites (Figure 8c). Upon expression of Tax1, each single mutant showed an approximate 50% decrease in promoter activity compared with the wild-type CD83 promoter (Figure 8d). Little, if any, response to Tax1 was observed for the mutant pCD83(-537 κBmt1/2)Luc carrying mutations at both sites (Figure 8d).

**Figure 9:**
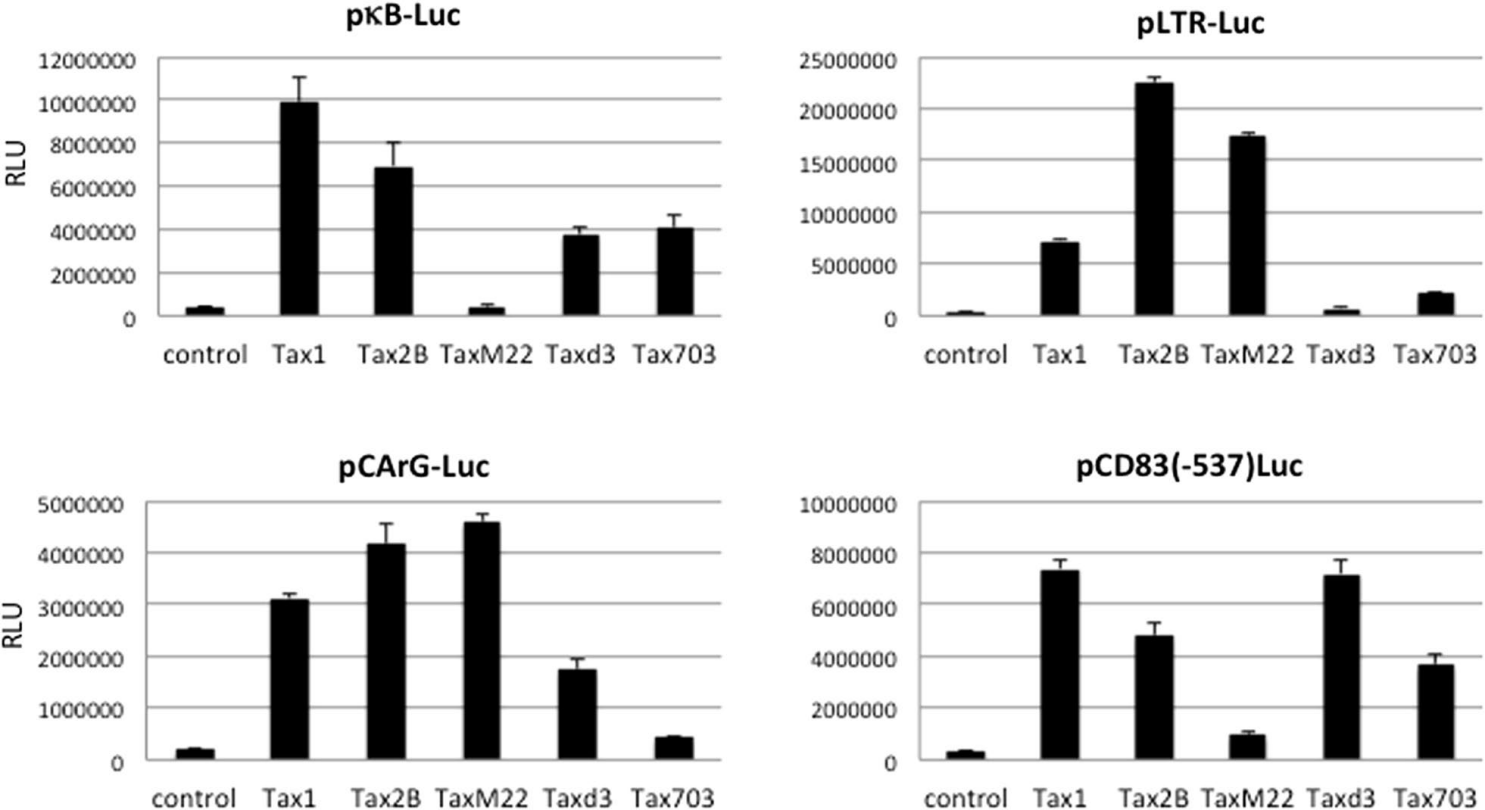
NF-κB-dependent activation of the CD83 promoter by Tax1. Jurkat cells were transfected with either Tax1, Tax2B, or its mutant plasmid along with the wild-type CD83 promoter reporter plasmid, cultured for 48 h, and then assayed for luciferase activity. The activities of the Tax expression plasmids utilized were confirmed based on their ability to induce cognate transcription factors under our experimental conditions. The results shown are the mean ± SE after normalization based on protein content.

The Tax1-mediated upregulation of CD83 expression via NF-κB activation was verified by reporter assays on a series of Tax1 mutants. All Tax1 mutants except for TaxM22, which lacks the NF-κB activation function, facilitated CD83 promoter activity (Figure 9). TaxM22 did not show significant induction of luciferase activity from pCD83(-537)Luc. Collectively, these results demonstrate that the two NF-κB binding sites are critical for the induction of CD83 expression in response to Tax1. HTLV-II Tax2B also upregulated CD83 promoter activity, consistent with cell surface expression of CD83 by the HTLV-II^+^ cells (Figure 2b).

### Stimulation of Tax1 expression and HTLV-I production by PGE2

**Figure 10:**
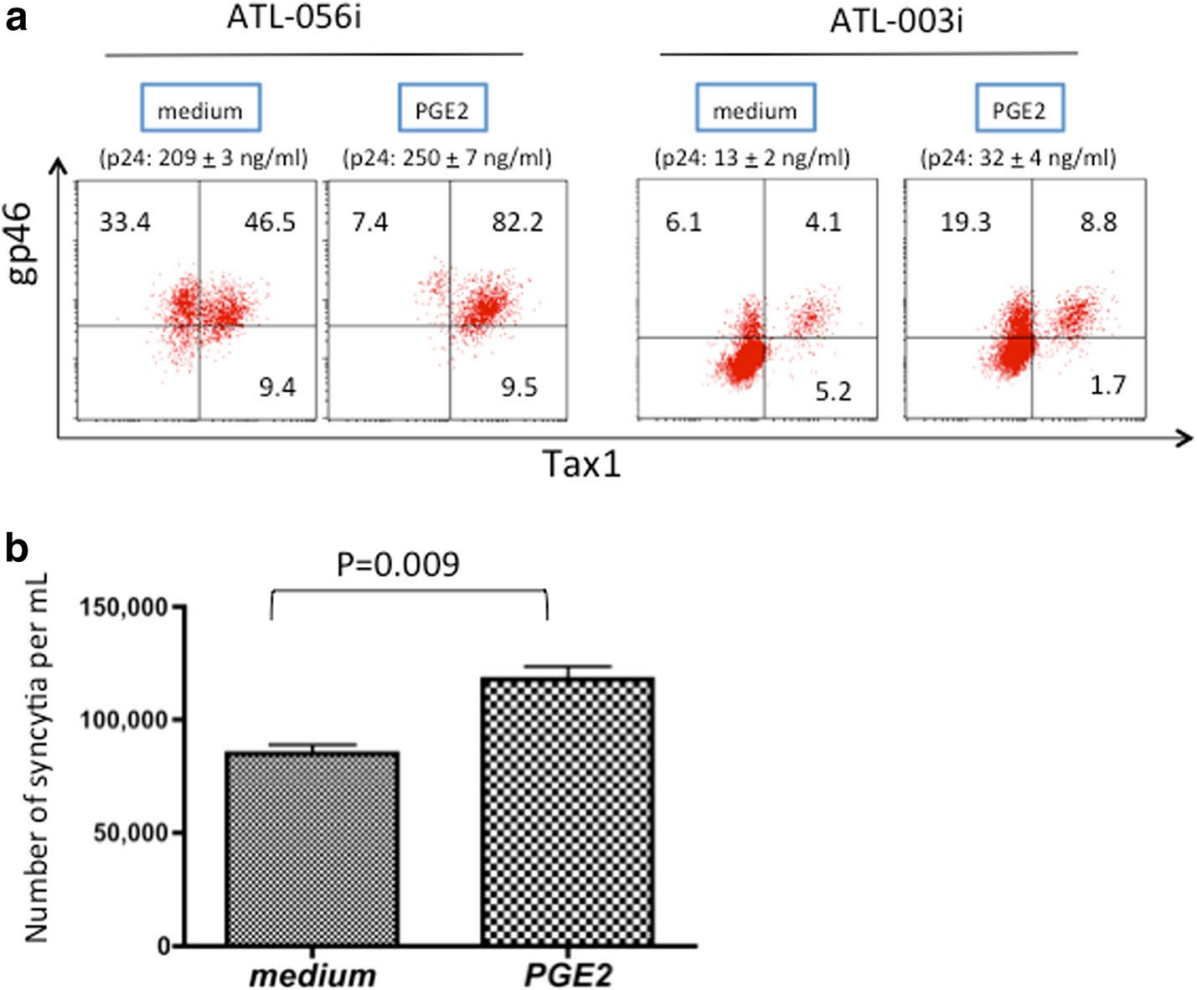
Enhancement of Tax1 expression and HTLV-I infectivity by prostaglandin E2 (PGE2). **a** ATL-patient-derived IL-2-dependent CD4^+^ T cell lines (ATL-056i and ATL-003i) were cultured in the presence or absence of 100 ng/ml PGE2 for 24 h and examined for the expression of Tax1 and HTLV-I envelope gp46 antigen by FCM. The levels of HTLV-I gag p24 released into the culture supernatants were determined by ELISA and are shown on the * top* of the dot plot. **b** ATL-056i cells suspended at 1 × 10^6^ cells/ml either untreated or treated with PGE2 for 24 h were co-cultured with an equal volume of Jurkat cells (1 × 10^6^ cells/ml) for an additional 18 h in triplicate wells. Then, the numbers of syncytia (per ml) were counted manually as described previously [26].

sCD83 has been reported to stimulate PGE2 production from human monocytes and to suppress T cell activation in vitro [15]. We thus tested the effects of PGE2 on HTLV-I expression in an ATL-derived HTLV-I^+^ T cell line (ATL-056). PGE2 induced marked expression of Tax1 (Figure 10a). In addition, we observed an increase in expression of the viral envelope protein gp46 and release of viral core protein p24 to the culture supernatants (Figure 10a). Figure 10b shows that PGE2 treatment increased the syncytium-inducing activity of ATL-056 cells, suggesting that PGE2 enhances cell-to-cell infectivity of HTLV-I.

## Discussion

In the present study, we showed for the first time that CD83 is expressed by Tax1^+^ cells in primary cultures of CD4^+^ T cells and HTLV-I- or HTLV-II-infected CD4^+^ or CD8^+^ T cell lines. Fresh ATL leukemic T cells express CD2, CD5, CD4, CD25, CD29, CD40RO, CD194, T cell receptor α/β, and HLA-DR with diminished CD3 expression [35], but they do not express Tax1. Cultures of primary CD4^+^ T cells from HTLV-I^+^ donors and HTLV-I^+^ T cell lines express additional cell surface markers including the T cell activation markers (CD40, CD80, and CD86), TNF/TNFR family members (GITR, 4-1BB, 4-1BBL, OX40, and OX40L), the chemokine receptors/interleukin receptors (CCR7 and CCR8), and a number of cell adhesion molecules, such as CD58/LFA3, CD54/ICAM-1, and VCAM-1. Some of those molecules are induced by Tax1 [36], and the present study adds CD83 to the list of members of the HTLV-I and -II Tax-inducible protein family.

Tax1 is a transcriptional modulator that lacks the ability to directly bind to DNA elements; rather, it modifies cellular transcription factor activity, in particular via NF-κB, CREB, and SRF. Among these factors, NF-κB attenuated by Tax1 is critically involved in immortalization and transformation. The present study demonstrates that Tax1-mediated induction of CD83 is absolutely dependent on the NF-κB pathway, similar to that reported for OX40 [34] and OX40L [36]. It has been reported that the Epstein-Barr virus latent membrane protein 1 (LMP1), which is known to activate the NF-κB pathway, also induces CD83 expression in human B lymphocytes, in which an NF-κB binding site in the CD83 promoter is responsive for LMP1-mediated activation [37]. Our present study revealed that another NF-κB binding site in the CD83 promoter was responsive to NF-κB activated by Tax1 in human T cells. It is of interest that the activation of NF-κB is insufficient for CD83 induction since a TL-Om1 cell line that contains a single copy of the HTLV-I provirus, but does not express Tax1, was negative for CD83 expression (Figure 2), while NF-κB in TL-Om1 is constitutively activated [38]. Thus, Tax1 expression may determine the promoter binding specificity and/or transcriptional regulation of NF-κB target genes such as CD83, OX40, and OX40L. Further studies are required to clarify the additional requirements for CD83 induction by Tax1.

The potential for CD83 induction by Tax1 in HTLV-I infections that leads to leukemogenesis or the onset of a neurological disorder, remains unknown. So far, CD83 modulation has been linked to virus escape from the T cell immune system. For example, human cytomegalovirus (HCMV) infection of DCs has been shown to inhibit T cell stimulation via release of sCD83 [39], and human herpes simplex virus (HSV) infection of mature DCs has been shown to downregulate the cell surface expression of CD83 by degradation of CD83 via the cellular proteasome [40]. Because the expression of either Tax1 or CD83 on CD4^+^ T cells is difficult to detect and monitor in the peripheral blood, even in acute ATL patients (Figure 1), Tax1-induced CD83 seems to be indirectly associated with the onset of ATL or HAM/TSP. However, observations of the steady presence of strong CTL and high levels of antibody responses specific for HTLV-I antigens in HTLV-I^+^-infected individuals indicate the persistent production of HTLV-I in vivo. In addition, an immunodeficiency was found in ATL patients or healthy carriers [27–31], and the inability of monocytes from HTLV-I^+^ donors to differentiate into mature DCs has been reported [32]. Integration of these results may provide some clues regarding the roles of CD83 in HTLV-I infection. First, Bates et al. recently reported that the homotypic interaction of mCD83 via cell-to-cell contact inhibits pro-inflammatory responses (including IL-12 production) induced by DCs [41]; accordingly, mCD83 induced on HTLV-I^+^ CD4^+^ T cells may interact with mCD83 on immuno-stimulated DCs in vivo and suppress IL-12 production, presumably resulting in inhibition of anti-HTLV-I Th1 responses. Second, sCD83 released from Tax1^+^ CD4^+^ T cells may interact with monocytes and stimulate the production of the pleotropic bioregulator PEG2. PEG2 has been reported to enhance HTLV-I gag p19 production from PBMC cultures of HTLV-I carriers [42] and to stimulate HTLV-I LTR in combination with CD3/CD28 activation in human T cell lines [43]. Consistent with these observations, we showed in the present study that PGE2 alone enhanced the expression of HTLV-I Tax1 and HTLV-I-mediated cell fusion (Figure 10). Therefore, it can be speculated that sCD83-induced PGE2 from monocytes stimulates HTLV-I production and the expansion of HTLV-I^+^ CD4^+^ T cells via the induction of Tax1 expression in a paracrine manner. However, our preliminary experiments failed to show that soluble recombinant CD83 actually enhances HTLV-I infection in vitro via production of PGE2 from primary monocytes. Further studies to test our hypotheses, including the link between T-cell activation by mCD83 and enhanced HTLV-I infection, are in progress.

Since normal human T cells express low to undetectable levels of CD83, high expression of both CD83 and OX40 on a single CD4^+^ T cell is a good marker of intracellular Tax1 expression. The sorting method for cells directly stained with anti-Tax antibody does not enable enrichment for live Tax1^+^ cells due to the required fixation and permeabilization of cells [44]. Thus, the present findings could provide a useful method for the enrichment of live Tax1^+^ T cells, as shown in Figure 7, and presumably will contribute to further studies on Tax1 function in the development and maintenance of HTLV-I-related diseases.

## Conclusions

The present study documents the potential role of HTLV-I Tax in the induction of CD83 cell surface expression and release of sCD83 in an NF-κB-dependent manner in human CD4^+^ T cells in vitro, which may indirectly suppress immune responses and promote HTLV-I infection in vivo in terms of virus transmission to new target cells and expansion and/or survival of infected T cell clones.

## Methods

### Reagents

The medium used throughout was RPMI 1640 medium (Sigma-Aldrich. Inc., St. Louis, MO, USA) supplemented with 10% fetal calf serum (FCS), 100 U/ml penicillin and 100 µg/ml streptomycin (hereinafter called RPMI medium). Mouse monoclonal antibodies (mAbs) specific for HTLV-I Tax (clone Lt-4), OX40 (clone B-7B5), and KLH (IgG3, clone KLH-3, Tanaka et al., unpublished) were purified in our laboratory from ascites fluids of CB.17-SCID mice carrying the appropriate hybridomas. The ascites fluid samples were subjected to ammonium sulfate precipitation followed by gel filtration with Superdex G-200 (GE Healthcare, Tokyo, Japan). mAbs were labeled with either fluorescein isothiocyanate (FITC) or HiLyte Fluor™ 647 using commercial labeling kits (Dojindo, Kumamoto, Japan) according to the manufacturer’s instructions. FITC-, PE- or PE-Cy7-labeled mouse mAbs against human CD3, CD4, CD8, CD14, CD19, CD56, and CD83 (clone H15e), and FITC-labeled goat anti-rat IgG, donkey anti-rabbit IgG, and HRP-labeled goat anti-rat IgG antibodies were purchased from BioLegend (Tokyo, Japan). Rabbit polyclonal IgG anti-rat CD83 antibody was obtained from Sino Biological (Beijing, China). Prostaglandin E2 (PGE2) was purchased from Sigma-Aldrich. Inc., (St. Louis, MO, USA).

### Cell cultures

The protocols for the use of human PBMCs and animals were approved by the Human IRB and the Institutional Animal Care and Use Committee (IACUC) on clinical and animal research of the University of the Ryukyus and Tokyo Medical and Dental University prior to the initiation of the study. All human samples were collected after obtaining written informed consent according to the Declaration of Helsinki. PBMCs were isolated from heparinized blood by a standard density gradient centrifugation [26]. HTLV-I-producing T cell lines were IL-2-independent T cell lines (MT-1, MT-2, and HUT102), IL-2-dependent CD4^+^ and CD8^+^ T cell lines derived from various HTLV-I^+^ donors (ATL patients, HAM/TSP patients, or asymptomatic HTLV-I carriers), and CD4^+^ T cell lines derived from normal PBMCs established by co-cultivation with mitomycin C-treated HTLV-I-producing cells [26]. The TL-Om1 cell line is an ATL-patient-derived IL-2-independent HTLV-I-infected T cell line not expressing the Tax antigen [38]. The HTLV-I^+^ rat T cell line was W7TM-1 [45]. The HTLV-I-negative human T cell lines were Jurkat, CEM, Molt-4, Molt-4/IIIB, and Jurkat-derived JPX-9, in which Tax1 can be induced by cultivation in the presence of 10 μM CdCl_2_ [46]. The HTLV-II^+^ human T cell line was Ton1 [47]. Cell lines were cultured in RPMI medium in the presence of 20 U/ml recombinant human IL-2, if necessary. For select experiments, human PBMCs were stimulated with 10 μg/ml phytohemagglutinin (PHA) (Wako, Japan) or immobilized anti-CD3 and anti-CD28 mAbs (BioLegend). Derivatives of 293T cells expressing human CD83 (293T/CD83) and its vector control (293T/CT) were established in our laboratory by transfection of 293T cells with human CD83 vector (pCAGIPuro/CD83) and empty vector (pCAGIPuro), respectively.

Syncytium induction assay was performed using a combination of ILT-M1 and Jurkat cells as described previously [26].

### Generation of a new anti-CD83 mAb

Hybridomas producing mAbs against human CD83 were generated from spleen cells of WKA rats immunized with recombinant human CD83-Fc fusion protein (R&D Systems, Inc., Cosmo Bio, Tokyo, Japan) by cell fusion with the SP2/0 myeloma cell line utilizing the Sendai virus cell fusion kit (GenomeONE, Cosmo Bio), and screened by ELISA using CD83-Fc-coated plates (0.1 μg/ml per well) and goat anti-rat IgG-HRP antibody (BioLegend). mAbs were further selected based on their abilities to stain CD83 expressed on 293T/CD83 cells but not 293T/CT cells. Western blots were performed as reported previously [48]. The anti-CD83 mAbs established by this screening included clones W83#4 and W83#8, both of which were rat IgG2b and applicable for flow cytometry. The clone W83#4, but not W83#8, competed in a binding assay with a commercially available mouse anti-human CD83 mAb clone H15e. The clone W83#8, but not W83#4 or H15e, was applicable for western blot analysis (data not shown).

### Flow cytometry (FCM), cell sorting, and ELISA

Phenotypic analyses of cells were carried out using polychromatic flow cytometry (FCM) as reported previously [26]. Briefly, live cells were Fc-blocked with 2 mg/ml pooled normal human IgG in FACS buffer [PBS containing 0.2% bovine serum albumin (BSA) and 0.1% sodium azide] for 10 min on ice, followed by incubation with fluorescent-dye labeled mAbs for 30 min. After washing with FACS buffer, the cells were fixed in 4% paraformaldehyde (PFA) in PBS for 5 min at room temperature followed by permeabilization and washing in FACS buffer containing 0.5% saponin and 1% BSA (Sigma). An aliquot of the stained cells was incubated with 0.1 μg/ml HiLyte Fluor™ 647-labeled anti-Tax1 antibody (clone Lt-4) for 30 min. Negative control cells were stained with HiLyte Fluor™ 647-labeled Lt-4 in the presence of 100 μg/ml unlabeled Lt-4. These cells were analyzed using a FACSCalibur (BD, Franklin Lakes, NJ, USA) and data obtained were analyzed using the Cell Quest software (BD).

To sort CD83 and OX40 double-positive and double-negative cell populations, IL-2-dependent T cell lines established from HTLV-I^+^ donors were stained with PE-Cy7-labeled anti-CD83 (clone H15e) and PE-labeled anti-OX40 (clone B-7B5) mAbs, and then subjected to sorting using a cell sorter SH800Z (Sony, Tokyo, Japan) equipped with appropriate filters for multi-color analysis in a dual semiconductor laser (488)/(639 nm) system.

sCD83 in culture supernatants and cell lysates were quantitated using a commercial ELISA kit (MyBiosource, San Diego, CA, USA). HTLV-I p24 production into the culture supernatants was determined by our in-house formulated ELISA [26].

### Plasmids

Expression plasmids for Tax1 (pMT-2Tax), Tax2B (pHβAP-r1-neo Tax2B), and Tax1 mutants were derived using the pHβAP-r-1-neo system with the human β-actin promoter [49, 50]. Tax1 mutants lacking the ability to activate NF-κB (TaxM22), CREB (Taxd3), and SRF (Tax703) in T cells have been described previously [50]. The luciferase reporter plasmids for the NF-κB site (pκB-Luc), CREB binding site (pLTR-Luc), and CArG box (pCArG-Luc) have been described elsewhere [51–53]. The CD83 reporter plasmid [pCD83(-537)Luc] was generated by insertion of a 578-bp fragment (−537 to +41 from the transcription start site [54]) amplified from human genomic DNA by PCR using forward (5′-acgctagccatggaaatctaacgcgccttt-3′) and reverse (5′-gtaagcttggctggagcgctgcgccgctgc-3′) primer pairs. The insert was cloned into the *Nhe*I and *Hin*dIII sites of the pGL3-basic vector. A series of 5′ deletion mutants of the human CD83 promoter was generated by PCR and inserted into pGL3-basic, yielding pCD83(-285)Luc, pCD83(-146)Luc, pCD83(-101)Luc, and pCD83(-29)Luc. The substitution mutants of the two possible NF-κB binding sites in the CD83 promoter were generated by PCR using the mutant primers, yielding pCD83(-537 κBmt1)Luc, pCD83(-537 κBmt2)Luc, and pCD83(-537 κBmt1/2)Luc with mutations at the NF-κB1 site, NF-κB2 site, and both sites, respectively (see Figure 8c, d).

### Infection with adenoviruses

Recombinant adenovirus for Tax1 (Ad-Tax1) [55] was generated with the ViraPower adenoviral expression system with the CAG promoter according to the supplier’s instructions. PBMCs and Jurkat cells were infected at an MOI of 100 or 10 plaque forming units (PFU)/cell, respectively, as previously described [56, 57].

### Quantitative PCR

Total RNA was extracted using Isogen (Nippon Gene Co. Ltd., Tokyo, Japan) according to the supplier’s protocol. First-strand cDNA was synthesized using the first-strand cDNA synthesis kit for reverse transcription-PCR (AMV; Roche, Tokyo, Japan). Quantification of CD83 gene expression was performed utilizing the forward (5′-gcgacgccggaggtgaaggtg-3′) and reverse (5′-tccccgagttgcagctggtagtgt-3′) primer pair using a LightCycler (Roche). The 18S rRNA primers were obtained from TaKaRa (Shiga, Japan).

### Reporter assay

Jurkat cells were transfected with Tax expression plasmids along with reporter plasmids by the DEAE-dextran method described previously [58]. Cell lysates were prepared for determination of luciferase activity using the luciferase assay system (Promega, Tokyo, Japan) according to the manufacture’s recommendations. Normalization of luciferase activity was performed against the cell lysate protein concentration that was measured by the DC Protein Assay Kit (Bio-Rad, Tokyo, Japan). The assays were repeated at least three times and the mean ± SE values are shown.

### Statistical analysis

A paired *t*-test was performed for statistical analysis. P-values of less than 0.05 were considered significant.

## Additional file

Additional file 1:
**Figure S1.** Western blot analysis of Tax1 expression in PBMCs from an ATL patient (ATL #5) before or after a one-day culture. Cell lysates were subjected to SDS-PAGE on a 5–20% gel, and blotted onto PVDF membranes. The membranes were incubated with either mouse anti-human Tax1 mAb (clone Lt-4) or IgG3 isotype control mAb (anti-KLH), followed by treatment with HRP-labeled goat anti-mouse IgG antibody.

## Abbreviations

HTLV-I: human T-cell leukemia virus type-I
Tax1: a transactivator from the X-gene region of HTLV-I
ATL: adult T-cell leukemia
FCM: flow cytometry
PBMCs: peripheral blood mononuclear cells
PHA: phytohemagglutinin
SDS-PAGE: SDS-polyacrylamide gel electrophoresis
MW: molecular weight
KLH: Keyhole limpet hemocyanin
