# Supplementary figures and images for: Human T-cell leukemia virus type-I Tax induces the expression of CD83 on T cells
Source: Retrovirology. 2015 Jul 1;12:56. doi: 10.1186/s12977-015-0185-1 (PMC4487981; doi:10.1186/s12977-015-0185-1)

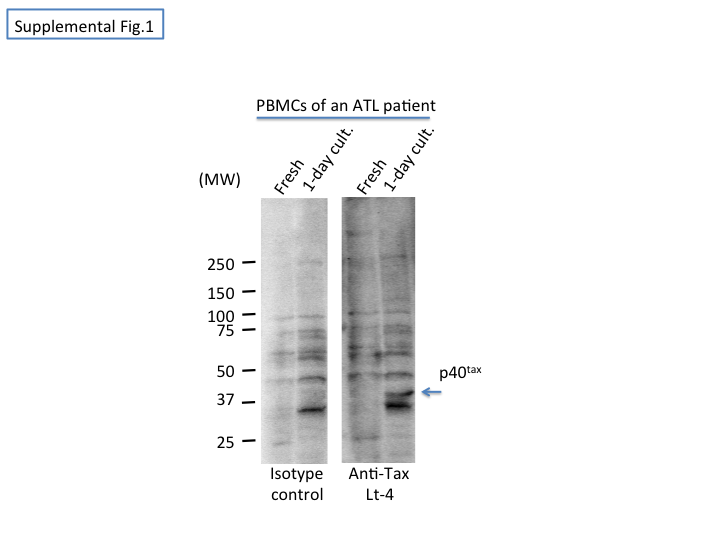

Supplement: Additional file 1: — Figure S1. Western blot analysis of Tax1 expression in PBMCs from an ATL patient (ATL #5) before or after a one-day culture. Cell lysates were subjected to SDS-PAGE on a 5–20% gel, and blotted onto PVDF membranes. The membranes were incubated with either mouse anti-human Tax1 mAb (clone Lt-4) or IgG3 isotype control mAb (anti-KLH), followed by treatment with HRP-labeled goat anti-mouse IgG antibody. [file 12977_2015_185_MOESM1_ESM.tiff]
